# Higher Infection of Dengue Virus Serotype 2 in Human Monocytes of Patients with G6PD Deficiency

**DOI:** 10.1371/journal.pone.0001557

**Published:** 2008-02-13

**Authors:** Yuan-Chang Chao, Ching-Shan Huang, Chun-Nan Lee, Sui-Yuan Chang, Chwan-Chuen King, Chuan-Liang Kao

**Affiliations:** 1 Department of Clinical Laboratory Sciences and Medical Biotechnology, College of Medicine, National Taiwan University (NTU), Taipei, Taiwan, Republic of China; 2 Department of Laboratory Medicine, NTU Hospital, Taipei, Taiwan, Republic of China; 3 Department of Medical Technology, Fooyin University, Kaohsiung, Taiwan, Republic of China; 4 Graduate Institute of Epidemiology, College of Public Health, NTU, Taipei, Taiwan, Republic of China; University of California Los Angeles, United States of America

## Abstract

The prevalence of glucose-6-phosphate dehydrogenase (G6PD) deficiency is high in Asia. An *ex vivo* study was conducted to elucidate the association of G6PD deficiency and dengue virus (DENV) infection when many Asian countries are hyper-endemic. Human monocytes from peripheral mononuclear cells collected from 12 G6PD-deficient patients and 24 age-matched controls were infected with one of two DENV serotype 2 (DENV-2) strains–the New Guinea C strain (from a case of dengue fever) or the 16681 strain (from a case of dengue hemorrhagic fever) with a multiplicity of infection of 0.1. The infectivity of DENV-2 in human monocytes was analyzed by flow cytometry. Experimental results indicated that the monocytes of G6PD-deficient patients exhibited a greater levels of infection with DENV-2 New Guinea C strain than did those in healthy controls [mean±SD:33.6%±3.5 (27.2%∼39.2%) vs 20.3%±6.2 (8.0%∼30.4%), *P<*0.01]. Similar observations were made of infection with the DENV-2 16681 strain [40.9%±3.9 (35.1%∼48.9%) vs 27.4%±7.1 (12.3%∼37.1%), *P<*0.01]. To our knowledge, this study demonstrates for the first time higher infection of human monocytes in G6PD patients with the dengue virus, which may be important in increasing epidemiological transmission and perhaps with the potential to develop more severe cases pathogenically.

## Introduction

Dengue virus (DENV), a member of the *Flaviviridae* family, has four serotypes of DENV-DENV-1, DENV-2, DENV-3 and DENV-4. Clinical manifestations of DENV infection range from asymptomatic to dengue fever (DF) and dengue hemorrhagic fever/dengue shock syndrome (DHF/DSS). About 100 million dengue cases occur around the world annually [1]. DF and DHF/DSS have emerged as the most important mosquito-borne viral diseases in tropical and subtropical countries, particularly in urban areas. DHF has expanded concomitantly in many geographical regions [2], [3]. However, the underlying mechanisms of DHF remain unknown.

The predominant target cells of DENV that are infected in humans have been identified as mononuclear phagocytes, monocytes, macrophage and dendritic cells [4]–[7]. These cells are responsible for disseminating the virus after its initial entry following the infection from mosquito bites. Since monocytes and macrophages are active phagocytic cells with cytoplasmic lysosomal components that can eliminate microorganisms [8], the interaction of the DENV with monocytes and macrophages may have detrimental effects on both virus and cells. Soluble mediators that are released from dengue virus-infected monocytes/macrophages strongly affect the biological characteristics of endothelial cells and the hematopoietic cell population, indicating that the interactions between dengue virus and monocytes/macrophages are important in the pathogenesis of DHF/DSS.

Glucose-6-phosphate dehydrogenase (G6PD) is an enzyme in the cytoplasm of all human cells [9]. G6PD deficiency that involves more than 300 allelic variants is one of the most inherited human disorders, as more than 400 million people are affected globally [10], [11]. The frequency of G6PD deficiency differs substantially among populations. About 7.5% of the global population carries one or two genes for G6PD deficiency. This proportion actually ranges from a maximum of 35% in parts of Africa, to 0.1% in Japan and parts of Europe [11]. High frequencies (6.0∼10.8%) of G6PD deficiency are also evident in Southeast Asia [12]. The overall prevalence of this deficiency is 2.1% in Taiwan [13]. The clinical manifestations of G6PD deficiency are neonatal jaundice, favism and acute haemolytic anemia [11]. Infection-induced hemolysis, involving many microbial agents, may be a common cause of clinically significant hemolytic anemia [14], [15] in G6PD-deficient patients, but its mechanism is unclear. Several studies have indicated that the abnormal function of leucocytes increases susceptibility to infection, such as by hepatitis A, in G6PD-deficient patients, causing more severe initial clinical presentations [9], [14], [16]–[18]. The association between G6PD deficiency and recurrent bacterial infection in children has been described elsewhere [18], [19]. A higher percentage of G6PD-deficient patients than non-G6PD-deficient patients has been associated with DHF/DSS (19.1%) in Thailand [20]. Epidemics of dengue in Taiwan have occurred frequently when imported cases have not been properly controlled [21]. The goals of this ex-vivo study are to find out whether monocytes from peripheral blood mononuclear cells (PBMC) of G6PD-deficient patients were more likely to be infected, regardless of the dengue virus strain. The results revealed that monocytes from PBMC of G6PD-deficient patients were more susceptible to DENV-2 infection with higher replication ability than those from healthy controls

## Results

### Growth curve of DENV-2 in human monocytes of healthy controls

**Figure 1:**
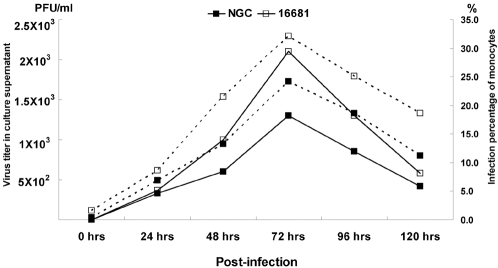
Growth of DENV-2 in human monocytes from one healthy control. Monocytes were infected with DENV-2 New Guinea C (NGC) and 16681 strains at an MOI of 0.1 for five days. The virus titer in the cell culture supernatants were analyzed by plaque assay (—) and the intracellular replication of virus in the infected cells were detected by flow cytometry ( ---- ).

**Figure 2:**
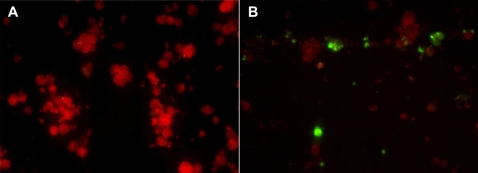
Detection of DENV in infected monocytes from one healthy control by using immunofluorescence staining. (A) Mock-infected cells. (B) DENV-2 infected monocytes. Monocytes were infected by DENV-2 New Guinea C strain at an MOI of 0.1 and then cultured for three days. Evans blue was added as a counter-stain to differentiate non-infected cells (shown by red color) from the dengue virus-positive infected cells (shown by green color).

PBMC monocytes from healthy controls obtained using the MACS monocyte isolation kit were infected with DENV-2 (New Guinea C or 16681 strain) at an MOI of 0.1. After those cells had been incubated in 5% CO_2 _at 37°C for five days, the infected monocytes and cell culture supernatants were harvested at various times post-infection. The quantitative measures of DENV-2 viral infection in monocytes were the percentage of cells to be infected, quantified by flow cytometry and the viral yields in the supernatant, determined by plaque assay (Figure 1). Flow cytometry indicated that human monocytes from healthy controls could be infected with DENV-2 and that the infections peaked on the third day post-infection for both New Guinea C and 16681 strains of DENV-2 (16681: 32.1% vs New Guinea C**:** 24.2% ). However, the percentages of human monocytes from the healthy controls that were infected with DENV-2 16681 strain exceeded that of those with DENV-2 New Guinea C strain from day one to day five post-infections. The infections of monocytes with DENV were also detected with indirect immunofluorescence stain (Figure 2A and 2B). Such infections were further verified by the virus yields of DENV-2 in the supernatants that are shown in Figure 1. Again, human monocytes infected with either New Guinea C or 16681 strains of DENV-2 exhibited similar growth patterns. The DENV-2 viral yield in cell culture supernatants also peaked three days post-infection and the viral yields of human monocytes from healthy controls that were infected with DENV-2 16681 also exceeded those that were infected with New Guinea C infection (2.1×10^3^ PFU/ml vs. 1.3×10^3^ PFU/ml). Restated, the viral yields from the supernatants of human monocytes from the healthy controls, measured by plaque assay, were very similar to the percentages of cells that were infected, as measured by flow cytometry. Based on this high correlation between the results of the two methods, flow cytometry was used to measure DENV-2 infectivity in infected monocytes in the following experiments.

### *Ex vivo* infection of DENV-2 in human monocytes from G6PD-deficient patients and healthy controls

**Figure 3:**
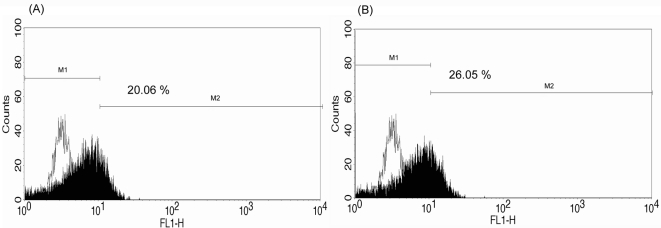
Flow cytometry analysis of DENV-2 infected human monocytes from one healthy control. New Guinea C strain (A) and 16681 strain (B) of DENV-2 were infected at an MOI of 0.1 and their infection levels on day three post-infection were measured by flow cytometry. Black: Percent DENV-2 infected cells. White: Percent mock-infected cells (background)

**Figure 4:**
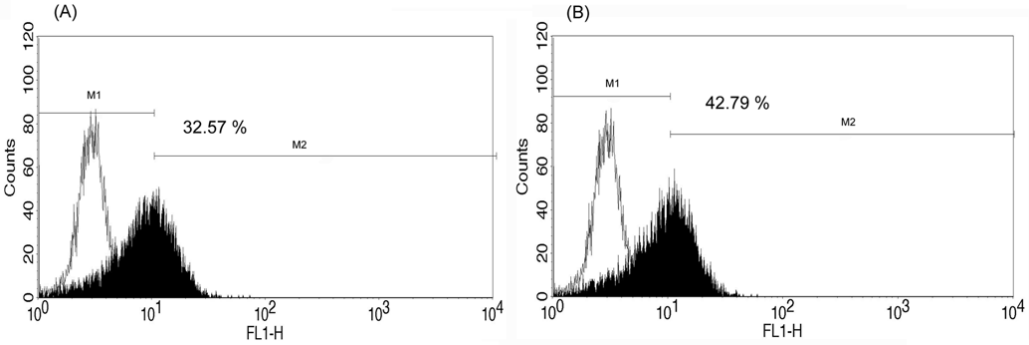
Flow cytometry analysis of DENV-2 infected human monocytes from one G6PD-deficient patient. Cells infected with New Guinea C strain (A) and 16681 strain (B) at an MOI of 0.1 and observed on day three post-infection. Black: Percent DENV-2 infected cells. White: Percent mock-infected cells (background)

Monocytes from G6PD-deficient patients and healthy controls were collected and separately infected with DENV-2. The infected cells were harvested and the infection for DENV-2 in human moncytes was analyzed by flow cytometry. Figures 3A and 3B plot the results obtained using the monocytes from one healthy donor infected with DENV-2 New Guinea C and 16681 strains, respectively. The percentage of positive cells was slightly higher in DENV-2 16681-infected cells (26.1%) than in DENV-2 New Guinea C-infected cells (20.1%). Figures 4A and 4B plot the results obtained using human PBMC monocytes from one G6PD-deficient patient who was infected with both of the aforementioned strains of DENV-2. The percentage of cells that were infected with the 16681 strain as well as the percentage of cells that were infected with the New Guinea C strains both exceeded the respective percentages of cells from the healthy controls that were infected (42.8% vs 26.1%, 32.6% vs 20.1%, respectively).

**Figure 5:**
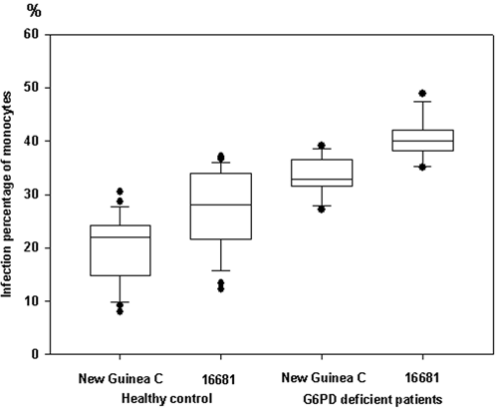
Box plot for comparison of the *ex vivo* infection percentages of DENV-2 (New Guinea C and 16681 strain) in monocytes of PBMC obtained from 12 G6PD-deficient patients and 24 healthy controls.

PBMC monocytes from 12 G6PD-deficient patients and 24 healthy controls were collected and infected with both strains of DENV-2 to investigate whether the above results concerning greater dengue viral infection in PBMC monocytes of G6PD-deficient patients than in those of healthy controls apply various G6PD patients. The results indicated that the mean percentage of human monocytes that were infected with New Guinea C in G6PD-deficient patients statistically significantly exceeded the percentage infection of those of the healthy controls [G6PD-deficient patients: 33.6±3.5% (27.2%∼39.2%) versus healthy controls: 20.3±6.2% (8.0%∼30.4%), *P<0.01*]. A similarly was obtained from cells that were infected with 16681. The mean percentages of human monocytes in G6PD-deficient patients and of those in healthy controls that were infected with DENV-2 16681 were 40.9±3.9% (35.1%∼48.9%) and 27.4±7.1% (12.3%∼37.1%), respectively (*P<0.01*). All of the 12 G6PD-deficient patients yielded similar results-with higher percentages of infected monocytes than were found in the 24 healthy donors (Figure 5). Comparing the infection capability of the two DENV-2 stains clearly reveals that more infected cells were present in 16681 than in New Guinea C-infected monocytes (*P<0.05)* that were collected from either G6PD-deficient patients or healthy controls.

### Detection of anti-DENV antibodies in G6PD-deficient patients using PRNT

Severe DENV infections have commonly observed in patients with secondary dengue viral infection in many South East Asian countries [1]–[3]. Plasma samples from all G6PD-deficient patients and healthy controls were tested in the presence of four DENV serotype-specific antibodies using PRNT to clarify whether the higher infection capability of DENV-2 in the monocytes of G6PD-deficient patients was caused by the secondary dengue viral infection. None of the four serotype specific DENV antibodies was detected in any of the 12 G6PD-deficient patients or 24 healthy controls. The results revealed that the higher infection percentage of DENV-2 in the monocytes of G6PD deficient-patients than in healthy people was not associated with prior DENV infections.

## Discussion

One of the important virological characteristics of severe dengue hemorrhagic fever (DHF) patients is elevated viral load [22]. The association of G6PD deficiency and microbial infections, such as acute viral hepatitis A, has been described [16]. This is the first work to demonstrate clearly that the PBMC monocytes obtained from G6PD-deficient patients, using an *ex vivo* culture system, were more easily infected with the two DENV-2 strains-(1) the New Guinea C strain from the DF patient or (2) the 16681 strain from the DHF patient than with those from healthy controls. This finding suggests that the high capability for DENV infection in human monocytes *in vivo* may increase viral yield, which may create more problems in the efficient clearance of the virus and thus increase the likelihood of a severe clinical outcome of dengue in G6PD-deficient patients. This suggestion is consistent with our earlier finding that DHF patients had higher viral loads even during the defervescence stage [22]. In Taiwan, the prevalence of G6PD deficiency in general population is about 2.1% [13]. When G6PD-deficient patients are infected with DENV, their higher viral load may increase the probability of transmission of the infection to others via infected mosquitoes, if mosquito breeding sites are not properly environmentally controlled [23]. In Thailand, the prevalence of G6PD deficiency in the general population is approximately 11% [20], explaining the higher prevalence of G6PD deficiency in DHF patients (19.1%) even though the study that supported this conclusion did not use a control group [20]. This conclusion also supports the public health implication that more efficient prevention and control of dengue is required in areas in which many G6PD-deficient patients live.

The mechanism of increased infection with DENV-2 in monocytes from G6PD-deficient patients remains unclear. The mutation in G6PD probably leads to granulocyte dysfunction, preventing the clearing of the infection at the first line of defense and thereby increasing susceptibility to DENV [24]. In fact, severe G6PD deficiency impairs respiratory burst activity and results in the generation of the less reactive oxygen species (ROS, including super-oxide anion, hydrogen peroxide and hydroxyl radical), causing abnormal function of the neutrophils and monocytes, as in HIV infection *in vitro* [25] and in ROS-deficient mice, which are extremely susceptible to infection [26]. Interestingly, ROS inhibited the RNA replication of hepatitis C virus, a member of the same family (*Flaviviridae*) as DENV, in human hepatoma cells [27]. Therefore, the greater infection of DENV in human monocytes from G6PD-deficient patients may be associated with the low activity of ROS in monocytes, which exhibit reduced activity for eliminating the invaded DENV-2 in infected cells.

The importance of host factors, such as human leukocyte antigen (HLA) and dendritic cell–specific intercellular adhesion molecule-3 grabbing nonintegrin (DC-SIGN) genes, in the pathogenesis of DENV infections has been documented [28], [29]. This study demonstrates that more attention should be paid to another host genetic factor (G6PD) and to co-morbidity on both individual and community levels when DENV infection has occurred. Effort must be made to conduct an international comparative case-control study in DHF endemic or hyper-endemic countries/areas with a range of prevalence of G6PD deficiency, by measuring the viral load and immunity in G6PD-deficient patients versus controls during the infecting process at various times following infection, as well as associated clinical outcomes. The results provide a new direction for elucidating the roles of host genetic factors in the pathogenesis of DENV-related diseases.

## Materials and Methods

### Study subjects

Twelve male patients with G6PD deficiency and 24 (18 male and eight female) age-matched healthy controls (with mean ages of 32.1±7.3 and 29.3±5.1****years respectively, *P = *0.2), as that shown in Table 1, participated in the study. G6PD deficiency was defined as enzyme activity of less than the reference value (4 IU/gHb), as measured using the ELISA method [30]. The mean G6PD activity of the G6PD-deficient patients (0.5±0.9 IU/gHb) was significantly less than that of the healthy controls (12.4±2.6 IU/gHb, *P<*0.01) (Table 1). All the 12 G6PD deficient male patients were confirmed to be G6PD deficient using the molecular-biology method as that has been described previously [30]: seven subjects carrying single-point mutation at nucleotide 1376 (G to T, Arg459Leu), four at nucleotide 1388 (G to A, Arg463His), and one at nucleotide 493 (A to G, Asn165Asp), respectively. All participants had written informed consent before their blood samples were withdrawn for further study. The protocol was also passed by the Ethical Committee of the College of Public Health at National Taiwan University. All 12 G6PD-deficient patients and the 21 healthy controls lived in northern Taiwan, with only sporadic dengue cases, but the three volunteers lived in southern Taiwan, where dengue epidemics occur more often.

**Table 1:** Gender and age distributions of study populations including G6PD-deficient patients and healthy controls

Study group	G6PD Enzyme activity*	No of Subjects	Age (Years) (mean±SD)
Healthy controls	12.38±2.63	16/8 (Males/Females)	29.3±5.09
G6PD deficiency Patients	0.45±0.91	12(Males)	32.1±7.34

* IU/g Hemoglobin (IU/gHb)

### Viruses

Two DENV-2 strains, the New Guinea C strain, isolated from a patient with dengue fever, and the 16681 strain, isolated from a patient with DHF, were grown from mosquito C6/36 cells in a growth medium of 50% Mitsumashi and Maramorsch Insect Medium (MMIM; Sigma, Saint Louis, Missouri, United States) plus 50% Dulbecco's modified Eagle's minimal essential medium (DMEM; GIBCO, Grand Island, NY, United States) plus 2% fetal bovine serum (FBS) at 28°C for 7 to 9 days. The viruses in the supernatants were harvested and stored at −80°C. The titers of the viruses were titrated in baby hamster kidney (BHK-21) cells using plaque assay.

### Isolation of human monocytes and cell culture

PBMCs from 10 ml peripheral blood samples from both G6PD-deficient patients and healthy controls were isolated in EDTA-containing tubes by density centrifugation with Ficoll-Hypaque. The mononuclear leukocytes recovered from the interface were washed twice by phosphate-buffered saline (PBS, pH 7.2) and suspended in RPMI 1640 medium with 10% FBS and penicillin/streptomycin [31]. Human monocytes were purified by depleting non-monocytes using a MACS® kit system (Miltenyi Biotec GmbH, Gladbach, Germany) by negative selection. Non-monocytes were indirectly magnetically labeled with a cocktail of biotin-conjugated mouse monoclonal antibodies against CD3, CD7, CD16, CD19, CD56, CD123 and CD235a, as primary labeling reagents, and anti-biotin monoclonal antibodies that were conjugated with MicroBeads, as secondary labeling reagents. The unlabeled monocytes passed through the column. The purity of these human monocytes (ranged from 91.4%–99.9%). The purified human monocytes were cultured in RPMI 1640 (GIBCO, Grand Island, NY, United States) medium with 10% FBS and 1% penicillin-streptomycin, as well as antimycotic (GIBCO, Grand Island, NY, United States), and incubated at 37°C in 5% CO_2_ incubator.

### *Ex vivo* infection of DENV-2

Human monocytes were each infected with one of the two strains of DENV-2, New Guinea C (DF strain) and 16681 (DHF strain), at a multiplicity of infection (MOI) of 0.1. Following absorption at 37°C for 3 hours, the monocytes were washed twice and suspended with medium. The infected monocytes were added in equal amounts to six-well plates, incubated at 37°C 5% CO_2_ for five days, and then quantified at various times post-infection by flow cytometry. Mock-infected cells were added to another plate as controls and were run simultaneously with the infected group to improve gating and the precision of measurement.

### Immunofluorescence stain

The infected and mock-infected monocytes were harvested and fixed on slides using cold acetone for 10 minutes. The monoclonal antibody (MAb) against DENV-2 (3H5) [32] was added to the fixed cells and then incubated at 37°C for 30 minutes. After the cells were washed, goat anti-mouse antibody conjugated with fluorescein isothiocyanate (FITC) (Kirkegaard & Perry Laboratories, United States) was added and incubated for another 30 minutes at 37°C. The results were observed under an immunofluorescence microscope for double confirmation before the samples were analyzed by flow cytometry.

### Flow cytometry

The human monocytes that were infected with the two DENV-2 strains (New Guinea C or 16681) and the mock infected monocytes were removed from the six-well plates, and washed twice with PBS (pH 7.2). The harvested cells were fixed and permeabilized with 4% paraformaldehyde and 0.2% sarponin. The permeabilized monocytes were washed and incubated with CD14-PE MAb (Becton Dickinson, San José, CA, United States) for 30 minutes at 4–8°C in the dark. Another mouse MAb (3H5) against DENV-2 was used to quantify the infected cells by incubating it with the tested human monocytes for 30 minutes at 4–8°C. The cells were then washed and incubated with FITC-labeled affinity-purified goat anti-mouse IgG (Kirkegaard & Perry Laboratories, United States) for 30 minutes at room temperature. Following incubation, the cells were washed twice in PBS (pH 7.2) and then analyzed using a FACScan flow cytometer (Becton Dickinson FACSCalibur System, United States). The mock-infected monocytes were run in parallel and served as negative controls. At least 10,000 cells were analyzed using a flow cytometer. Data were analyzed using Cell Quest software (Becton Dickinson, San José, CA, United States). The percentage of positive cells and the average fluorescence intensities were determined from FITC fluorescence histograms using a region that was defined based on the analysis of the mock-infected control cells).

### Plaque Reduction Neutralization Test (PRNT)

PRNT was performed to detect the serotypes of DENV antibodies that had been infected in the past, from plasma samples collected from G6PD-deficient patients, with reference to healthy controls. Four prototype DENVs (DENV-1:Hawaii, DENV-2:New Guinea C, DENV-3:H87 and DENV-4:H241, all obtained from Dr. Duane Gubler at the U.S. Centers for Disease Control and Prevention, Atlanta, Georgia. United States) were used in PRNT. Briefly, the plasma was inactivated at 56°C for 30 minutes. The four-fold diluted (1:10 ∼1:640) plasma and viruses were mixed well in equal amounts in the 96-well plates and incubated at 4°C overnight. The mixture was added to the BHK-21 cells that had been grown in the 24-well plates and incubated at 37°C 5% CO_2_ for one week. After the cells were fixed with 5% crystal violet, the plaques were counted. The serotiters of the neutralization antibody were determined using a 75% reduction from the number of plaques of the virus control as a cut-off point. The virus titer was expressed as the number of plaques that formed unit per ml (PFU/ml).

### Statistical Tests

The Student's T test was used to analyze the differences in means of percentages of DENV-2 positive infected monocytes between the patients with G6PD and age-matched healthy controls.
